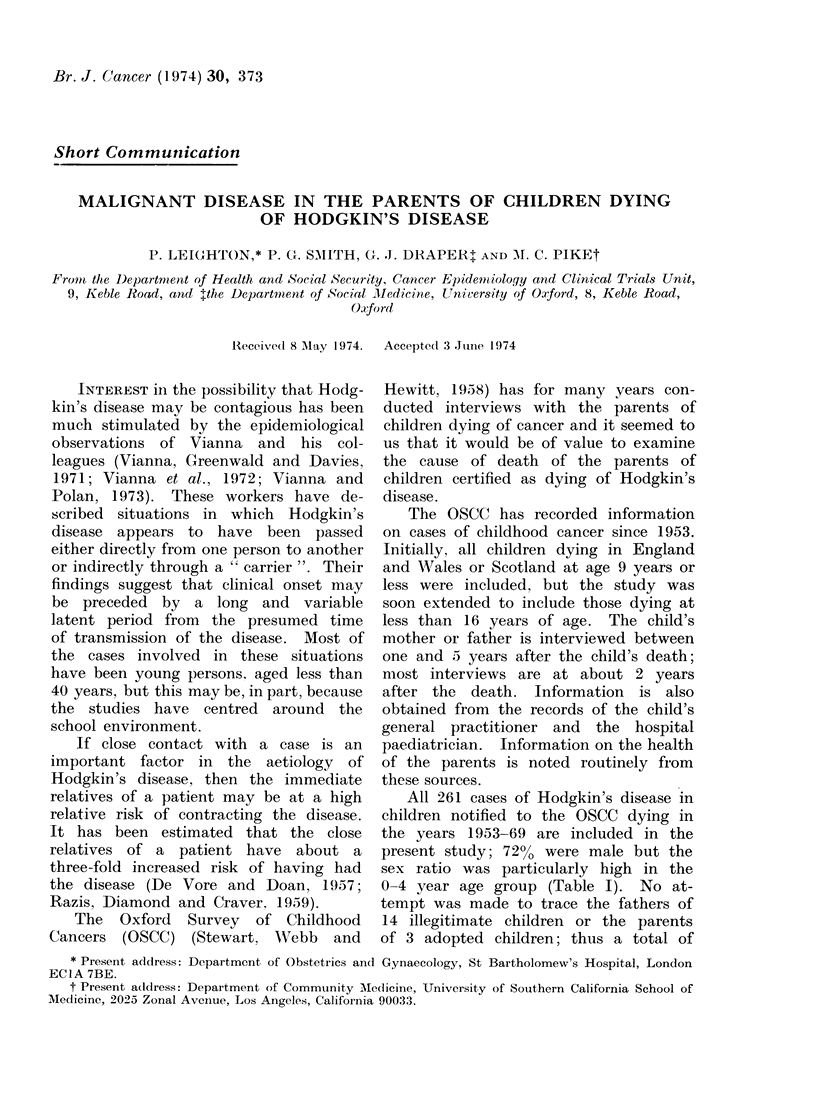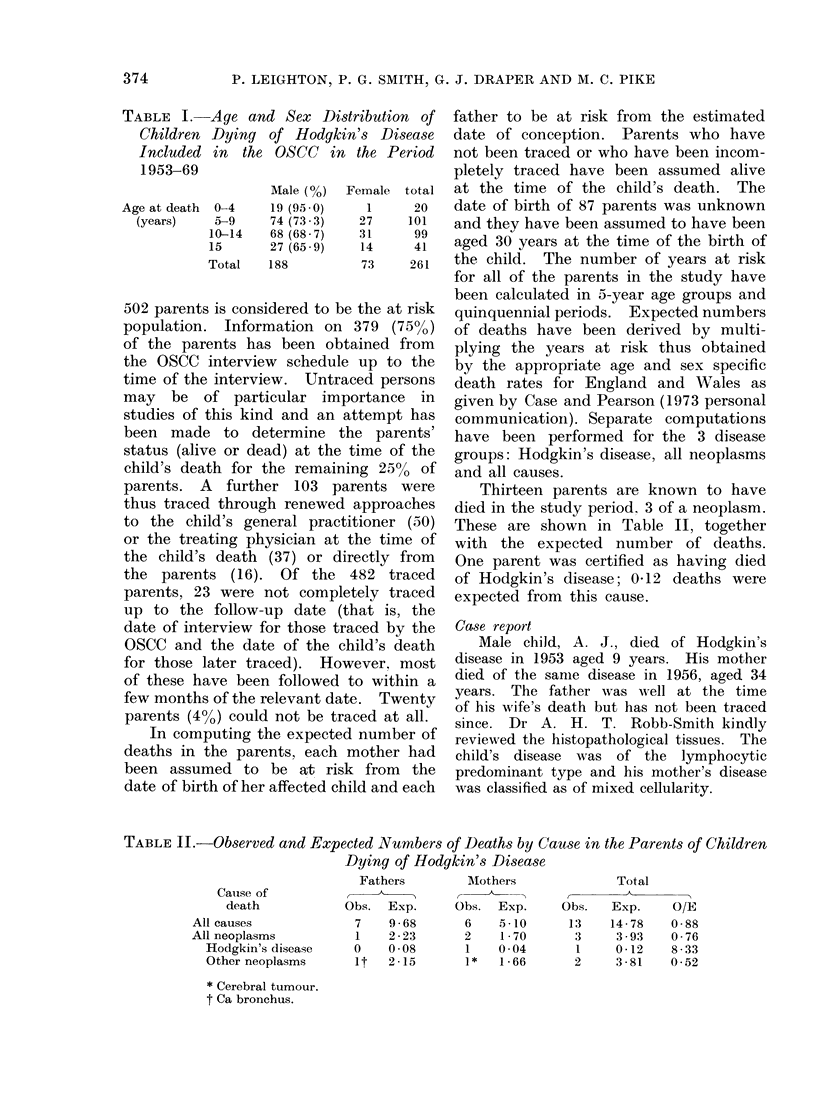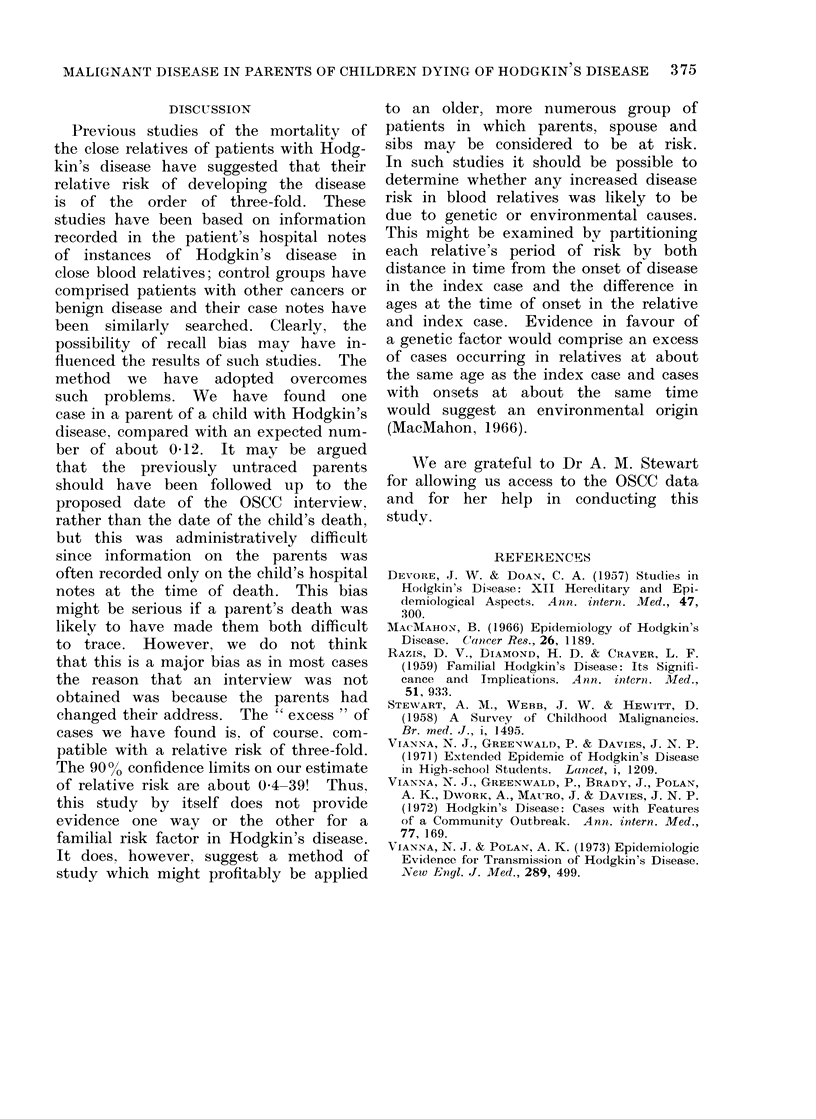# Malignant disease in the parents of children dying of Hodgkin's disease.

**DOI:** 10.1038/bjc.1974.208

**Published:** 1974-10

**Authors:** P. Leighton, P. G. Smith, G. J. Draper, M. C. Pike


*Br. J. Cancer* (1974) **30**, 373

***Short Communication***

### MALIGNANT DISEASE IN THE PARENTS OF CHILDREN DYING OF HODGKIN'S DISEASE

P. LEIGHTON,* P. G. SMITH, G. J. DRAPER‡ AND M. C. PIKE†

*From the Department of Health and Social Security, Cancer Epidemiology and Clinical Trials Unit, 9, Keble Road, and ‡the Department of Social Medicine, University of Oxford, 8, Keble Road, Oxford*

Received 8 May 1974. Accepted 3 June 1974

INTEREST in the possibility that Hodgkin's disease may be contagious has been much stimulated by the epidemiological observations of Vianna and his colleagues (Vianna, Greenwald and Davies, 1971; Vianna *et al.*, 1972; Vianna and Polan, 1973). These workers have described situations in which Hodgkin's disease appears to have been passed either directly from one person to another or indirectly through a " carrier ". Their findings suggest that clinical onset may be preceded by a long and variable latent period from the presumed time of transmission of the disease. Most of the cases involved in these situations have been young persons, aged less than 40 years, but this may be, in part, because the studies have centred around the school environment.

If close contact with a case is an important factor in the aetiology of Hodgkin's disease, then the immediate relatives of a patient may be at a high relative risk of contracting the disease. It has been estimated that the close relatives of a patient have about a three-fold increased risk of having had the disease (De Vore and Doan, 1957; Razis, Diamond and Craver, 1959).

The Oxford Survey of Childhood Cancers (OSCC) (Stewart, Webb and Hewitt, 1958) has for many years conducted interviews with the parents of children dying of cancer and it seemed to us that it would be of value to examine the cause of death of the parents of children certified as dying of Hodgkin's disease.

The OSCC has recorded information on cases of childhood cancer since 1953. Initially, all children dying in England and Wales or Scotland at age 9 years or less were included, but the study was soon extended to include those dying at less than 16 years of age. The child's mother or father is interviewed between one and 5 years after the child's death; most interviews are at about 2 years after the death. Information is also obtained from the records of the child's general practitioner and the hospital paediatrician. Information on the health of the parents is noted routinely from these sources.

All 261 cases of Hodgkin's disease in children notified to the OSCC dying in the years 1953–69 are included in the present study; 72% were male but the sex ratio was particularly high in the 0–4 year age group (Table I). No attempt was made to trace the fathers of 14 illegitimate children or the parents of 3 adopted children; thus a total of

* Present address: Department of Obstetrics and Gynaecology, St Bartholomew's Hospital, London EC1A 7BE.

† Present address: Department of Community Medicine, University of Southern California School of Medicine, 2025 Zonal Avenue, Los Angeles, California 90033.

TABLE I.—*Age and Sex Distribution of Children Dying of Hodgkin's Disease Included in the OSCC in the Period 1953–69*

| | | Male (%) | Female | total |
|---|---|---|---|---|
| Age at death | 0–4 | 19 (95·0) | 1 | 20 |
| (years) | 5–9 | 74 (73·3) | 27 | 101 |
| | 10–14 | 68 (68·7) | 31 | 99 |
| | 15 | 27 (65·9) | 14 | 41 |
| | Total | 188 | 73 | 261 |

502 parents is considered to be the at risk population. Information on 379 (75%) of the parents has been obtained from the OSCC interview schedule up to the time of the interview. Untraced persons may be of particular importance in studies of this kind and an attempt has been made to determine the parents' status (alive or dead) at the time of the child's death for the remaining 25% of parents. A further 103 parents were thus traced through renewed approaches to the child's general practitioner (50) or the treating physician at the time of the child's death (37) or directly from the parents (16). Of the 482 traced parents, 23 were not completely traced up to the follow-up date (that is, the date of interview for those traced by the OSCC and the date of the child's death for those later traced). However, most of these have been followed to within a few months of the relevant date. Twenty parents (4%) could not be traced at all.

In computing the expected number of deaths in the parents, each mother had been assumed to be at risk from the date of birth of her affected child and each father to be at risk from the estimated date of conception. Parents who have not been traced or who have been incompletely traced have been assumed alive at the time of the child's death. The date of birth of 87 parents was unknown and they have been assumed to have been aged 30 years at the time of the birth of the child. The number of years at risk for all of the parents in the study have been calculated in 5-year age groups and quinquennial periods. Expected numbers of deaths have been derived by multiplying the years at risk thus obtained by the appropriate age and sex specific death rates for England and Wales as given by Case and Pearson (1973 personal communication). Separate computations have been performed for the 3 disease groups: Hodgkin's disease, all neoplasms and all causes.

Thirteen parents are known to have died in the study period, 3 of a neoplasm. These are shown in Table II, together with the expected number of deaths. One parent was certified as having died of Hodgkin's disease; 0·12 deaths were expected from this cause.

*Case report*

Male child, A. J., died of Hodgkin's disease in 1953 aged 9 years. His mother died of the same disease in 1956, aged 34 years. The father was well at the time of his wife's death but has not been traced since. Dr A. H. T. Robb-Smith kindly reviewed the histopathological tissues. The child's disease was of the lymphocytic predominant type and his mother's disease was classified as of mixed cellularity.

TABLE II.—*Observed and Expected Numbers of Deaths by Cause in the Parents of Children Dying of Hodgkin's Disease*

| Cause of death | Fathers | | Mothers | | Total | | |
|---|---|---|---|---|---|---|---|
| | Obs. | Exp. | Obs. | Exp. | Obs. | Exp. | O/E |
| All causes | 7 | 9·68 | 6 | 5·10 | 13 | 14·78 | 0·88 |
| All neoplasms | 1 | 2·23 | 2 | 1·70 | 3 | 3·93 | 0·76 |
| Hodgkin's disease | 0 | 0·08 | 1 | 0·04 | 1 | 0·12 | 8·33 |
| Other neoplasms | 1† | 2·15 | 1* | 1·66 | 2 | 3·81 | 0·52 |

* Cerebral tumour.

† Ca bronchus.

### DISCUSSION

Previous studies of the mortality of the close relatives of patients with Hodgkin's disease have suggested that their relative risk of developing the disease is of the order of three-fold. These studies have been based on information recorded in the patient's hospital notes of instances of Hodgkin's disease in close blood relatives; control groups have comprised patients with other cancers or benign disease and their case notes have been similarly searched. Clearly, the possibility of recall bias may have influenced the results of such studies. The method we have adopted overcomes such problems. We have found one case in a parent of a child with Hodgkin's disease, compared with an expected number of about 0·12. It may be argued that the previously untraced parents should have been followed up to the proposed date of the OSCC interview, rather than the date of the child's death, but this was administratively difficult since information on the parents was often recorded only on the child's hospital notes at the time of death. This bias might be serious if a parent's death was likely to have made them both difficult to trace. However, we do not think that this is a major bias as in most cases the reason that an interview was not obtained was because the parents had changed their address. The " excess " of cases we have found is, of course, compatible with a relative risk of three-fold. The 90% confidence limits on our estimate of relative risk are about 0·4–39! Thus, this study by itself does not provide evidence one way or the other for a familial risk factor in Hodgkin's disease. It does, however, suggest a method of study which might profitably be applied to an older, more numerous group of patients in which parents, spouse and sibs may be considered to be at risk. In such studies it should be possible to determine whether any increased disease risk in blood relatives was likely to be due to genetic or environmental causes. This might be examined by partitioning each relative's period of risk by both distance in time from the onset of disease in the index case and the difference in ages at the time of onset in the relative and index case. Evidence in favour of a genetic factor would comprise an excess of cases occurring in relatives at about the same age as the index case and cases with onsets at about the same time would suggest an environmental origin (MacMahon, 1966).

We are grateful to Dr A. M. Stewart for allowing us access to the OSCC data and for her help in conducting this study.

### REFERENCES

DEVORE, J. W. & DOAN, C. A. (1957) Studies in Hodgkin's Disease: XII Hereditary and Epidemiological Aspects. *Ann. intern. Med.*, **47**, 300.

MACMAHON, B. (1966) Epidemiology of Hodgkin's Disease. *Cancer Res.*, **26**, 1189.

RAZIS, D. V., DIAMOND, H. D. & CRAVER, L. F. (1959) Familial Hodgkin's Disease: Its Significance and Implications. *Ann. intern. Med.*, **51**, 933.

STEWART, A. M., WEBB, J. W. & HEWITT, D. (1958) A Survey of Childhood Malignancies. *Br. med. J.*, i, 1495.

VIANNA, N. J., GREENWALD, P. & DAVIES, J. N. P. (1971) Extended Epidemic of Hodgkin's Disease in High-school Students. *Lancet*, i, 1209.

VIANNA, N. J., GREENWALD, P., BRADY, J., POLAN, A. K., DWORK, A., MAURO, J. & DAVIES, J. N. P. (1972) Hodgkin's Disease: Cases with Features of a Community Outbreak. *Ann. intern. Med.*, **77**, 169.

VIANNA, N. J. & POLAN, A. K. (1973) Epidemiologic Evidence for Transmission of Hodgkin's Disease. *New Engl. J. Med.*, **289**, 499.